# Asynchronous collision integrators: Explicit treatment of unilateral contact with friction and nodal restraints

**DOI:** 10.1002/nme.4516

**Published:** 2013-07-03

**Authors:** Sebastian Wolff, Christian Bucher

**Affiliations:** Forschungsbereich für Baumechanik und Baudynamik, Technische Universität WienKarlsplatz 13/E2063, 1040 Wien, Austria

**Keywords:** contact, Hamiltonian, nonlinear dynamics, time integration, explicit, variational methods

## Abstract

This article presents asynchronous collision integrators and a simple asynchronous method treating nodal restraints. Asynchronous discretizations allow individual time step sizes for each spatial region, improving the efficiency of explicit time stepping for finite element meshes with heterogeneous element sizes. The article first introduces asynchronous variational integration being expressed by drift and kick operators. Linear nodal restraint conditions are solved by a simple projection of the forces that is shown to be equivalent to RATTLE. Unilateral contact is solved by an asynchronous variant of decomposition contact response. Therein, velocities are modified avoiding penetrations. Although decomposition contact response is solving a large system of linear equations (being critical for the numerical efficiency of explicit time stepping schemes) and is needing special treatment regarding overconstraint and linear dependency of the contact constraints (for example from double-sided node-to-surface contact or self-contact), the asynchronous strategy handles these situations efficiently and robust. Only a single constraint involving a very small number of degrees of freedom is considered at once leading to a very efficient solution. The treatment of friction is exemplified for the Coulomb model. Special care needs the contact of nodes that are subject to restraints. Together with the aforementioned projection for restraints, a novel efficient solution scheme can be presented. The collision integrator does not influence the critical time step. Hence, the time step can be chosen independently from the underlying time-stepping scheme. The time step may be fixed or time-adaptive. New demands on global collision detection are discussed exemplified by position codes and node-to-segment integration. Numerical examples illustrate convergence and efficiency of the new contact algorithm. Copyright © 2013 The Authors. *International Journal for Numerical Methods in Engineering* published by John Wiley & Sons, Ltd.

## 1. INTRODUCTION

### Asynchronous integration

The main disadvantage of explicit time integration is its conditional stability, that is, it becomes unstable if the time step exceeds a certain threshold. In many applications only a small number of finite elements of very small size and/or with stiff material properties are responsible for the small size of this critical time step.

A popular approach to overcome this problem are multiple time stepping integrators. One line of development starts with mixed methods by using implicit and explicit time stepping for different domains 1, another line uses different time step sizes known as subcycling 2. Multiple time stepping creates configurations at discrete times *t*_*k*_ = *k* ⋅ Δ*t* where all nodal displacements and velocities are synchronous in time. Some parts of the structure are substepped using a smaller time step at configurations in-between where not the whole system is synchronous. These smaller time steps are obtained by bisection, integer ratios or non-integer ratios 3–5.

A generalization of the symplectic-momentum multiple-time stepping scheme r-RESPA 6 are asynchronous variational integrators (AVIs) 7–12. Therein, time step sizes are individually assigned to each finite element at arbitrary ratios. It is, therefore, generally not possible to obtain configurations in time where all finite elements are evaluated at the same ‘synchronous’ time. The time stepping scheme can be derived as a variational integrator 13 and, thus, is symplectic 14 and momentum preserving 15. Convergence can be proved for linear elasticity 11. Reliable stability criteria are difficult to find. A stability analysis was exemplified for a single degree of freedom system with two asynchronous potential functions in 12.

The conditional stability of explicit integrators is the reason why implicit time stepping schemes are often preferred. For linear systems implicit schemes may be unconditionally stable allowing arbitrarily large time steps. In nonlinear dynamics, symplectic implicit schemes are also only conditionally stable. Even energy-conserving time stepping schemes may become unstable when applied to nonlinear systems 16. Benes and Matous 17, 18 have shown computationally that the critical time step for their implicit asynchronous integrators drops sharply but saturates at a level within the range of reasonable engineering accuracy. They also show that the critical time step for explicit asynchronous integrators improves over the synchronous case. This result agrees with 19 on improving stability by mollified impluses in r-RESPA and the references therein. As noted in 12, however, there may be time step sizes below the numerically assessed stability limit where asynchronous schemes are unstable.

The motivation of this article is to extend the idea behind explicit asynchronous integration to the context of explicit contact/impact dynamics and to explore the potential computational savings by this approach.

### Contact dynamics

Unilateral constraints often arise in contact/impact problems where an impenetrability condition 20 must be satisfied that is represented by an inequality condition. There are generally two approaches to dynamic discretization of the constraint: enforcing impenetrability at discrete points in time or enforcing the time derivative of impenetrability (persistency condition) being zero.

The enforcement of the persistency condition is known as Laursen–Chawla algorithm 21. Therein, an analysis of the generalized-alpha and Newmark methods leads to the observation that energy conservation is tied to the persistency condition. An application to the augmented Lagrangian and penalty method is presented that are either conservative or dissipative and allow small penetrations. The algorithm was later combined with the impenetrability condition whereby energy conservation is restored by an additional velocity update 22, 23. A variational treatment based on a discrete version of the classical principle of Hamilton is given by 24 who introduce the collision time as additional degree of freedom in explicit integrators and perform a velocity jump that is equivalent to enforcing the persistency condition. This leads to a nonsmooth trajectory. The approach was further simplified in 25 introducing decomposition contact response (DCR) that is a non-iterative treatment at discrete points in time extending to inelastic and frictional contact problems.

### Asynchronous collisions

This article presents contact algorithms to explicit asynchronous simulation of structural dynamics. When handling contact problems in explicit dynamics, there generally exist two approaches: penalty based and Lagrange multiplier methods. Penalty methods are simple to implement, and penalty forces can be computed efficiently. But they are inaccurate allowing penetrations and may affect the critical time step. A penalty approach to asynchronous contact was presented in 26. Lagrange multiplier methods, on the other hand, often lead to iterative procedures. The possible large number of highly nonlinear constraints reduces the efficiency. Furthermore, redundant constraints may appear leading to singular systems of equations.

The application of an asynchronous collision integrator may eliminate some problems arising in Lagrange multiplier methods. Let the individual contact constraints be enforced at asynchronous times. If each spatial constraint is considered individually, the system of equations is simplified by two factors: (1) there is only a single constraint to be enforced at one time; and (2) furthermore, only a limited number of degrees of freedom is affected. The size of the equation system is, therefore, very small. By application of DCR, the equations are linear and the constraints can be enforced non-iteratively. The operation only modifies the momentum and can, thus, be interpreted in terms of a kick operator of an asynchronous variational integration algorithm. Because each constraint is considered individually without affecting the critical time step, one may chose the time step size between two contact corrections according to local accuracy conditions, such as relative velocities and finite element sizes. The formulation of the adaptive time step is much easier than for the potential energy where either symplecticity must be restored by additive terms that may lead to iterative schemes even for explicit methods 27 or where instabilities occur because of unsolvable equations 28.

### Objectives and outline

One objective of this article is to develop an efficient implementation of nodal restraint conditions for AVI. The main focus is, however, to derive a suitable explicit collision integrator within asynchronous variational integration. In particular, its efficient implementation and its coupling with nodal restraints is the main contribution of this article.

The outline is as follows: Section 2 recalls the basic ideas of AVI. Section 3 explains the notation used in this article by interpreting AVI as a sequence of drifts with constant motion and a set of events that modify the velocity asynchronously. The novel implementation of nodal restraints will be derived in Section 4 on the basis of the variational RATTLE method. Implications of the asynchronous approach to global contact detection algorithms are briefly discussed in Section 5 by the example of position codes and node-to-surface integration. The asynchronous collision integrator is finally presented in Section 6. For completeness, the decomposition contact response is briefly shown. Subsequently, three novel and efficient solution algorithms of DCR in the context of AVI are formulated: normal contact, normal contact with nodes being subject to restraint conditions and normal contact with friction being exemplified by the Coulomb model. Three configurations of AVI are presented in Section 7 introducing a synchronous setting and asynchronous settings with fixed and variable step sizes. Numerical examples illustrate convergence and efficiency of the new contact algorithm in Section 8.

### Symbols and typography

The notation in Sections 2 to 4 follows the lines of articles on variational integrators, For example 8, 9, 12, 24, 28, 29. Matrices, vectors, and scalars are characterized by non-bold letters, that is, all generalized coordinates are collected in a vector *q*, whereas its conjugate momenta are defined as *j*. Sections 5 to 7 are related to the spatial and temporal contact formulation and introduce the notation that is more familiar to most engineers in mechanics, that is, small bold letters for vectors, large bold letters for matrices. The generalized coordinates then specialize to the vectors of nodal displacements **u** whereas one often uses the vector of velocities **v** instead of momenta.

## 2. ASYNCHRONOUS VARIATIONAL INTEGRATION

Assume that the total potential energy is obtained by some additive composition


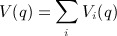
(1)

with a vector of generalized coordinates *q*. In FEM, the potential energy *V* (*q*) is generally composed by the sum of weighted strain energy density functions at numerical integration points. Usually, the individual potentials *V*_*i*_(*q*) are the contributions of single finite elements to the strain energy. The mass matrix *M* is assumed to be diagonal with *M* = diag(*m*_*A*_) introducing the nodal mass 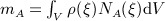.

Standard time stepping schemes evaluate the composition *V* (*q*) in one step, that is, the potentials *V*_*i*_(*q*) and their derivatives are computed at synchronous times. Asynchronous integration aims at evaluating the individual potentials at separate times. AVIs can be configured such that they resemble standard and multiple time stepping schemes. Discrete times when all potentials are synchronous do not, however, exist in general.

Assigned to each potential *V*_*i*_ a sequence of times 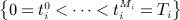. Another sequence is created by inserting all times 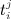 into a unique and sorted set that then contains all system times 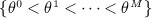. The solution trajectory is obtained by a piecewise linear interpolation of the generalized coordinates *q*(*t*) along their supports at the system times *θ*_*k*_. Continuity of *q*(*t*) at *θ*_*k*_ is enforced by Lagrange multipliers *j*_*k*_ that can be identified as discrete momenta. Define a function 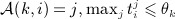 that determines the index *j* of time 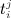 where the potential *V*_*i*_ has been evaluated most recently prior system time *θ*_*k*_. The function 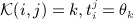 returns the index *k* on the total time scale *θ* for a given index pair (*i*,*j*) defining the potential *V*_*i*_ and the potential time index *j*.

The asynchronous time integrator is then given through the time stepping scheme


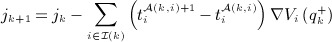
(2)


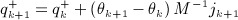
(3)

see 12, 30 for a detailed illustration of the formulation and notation. In words, at time *θ*_*k*_ one determines all potentials that are part of the set 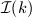, that is, which are active at this time. The modification of the momentum is identical to symplectic synchronous Euler except that the time step sizes, which scale the contributions of all active potentials *V*_*i*_, are not identical to the size of the time element (*θ*_*k* + 1_ − *θ*_*k*_) but are the time steps of the potentials 
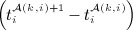
. The trajectory of the generalized coordinates within the time element is characterized by a constant motion by using the modified momentum *j*_*k* + 1_.

## 3. SEQUENCE OF KICKS AND DRIFTS

**Figure 1:**
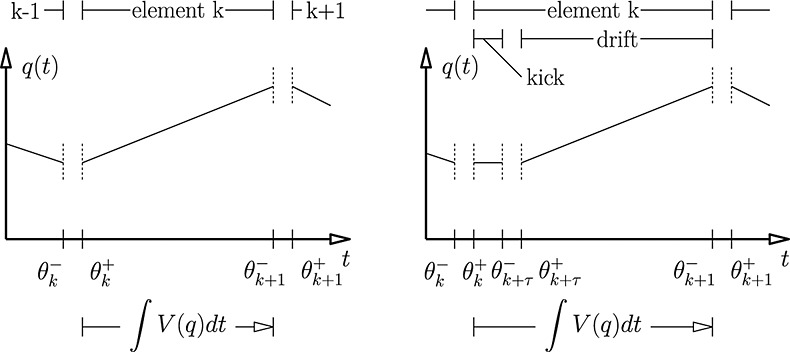
Variational kick and drift elements. Element *k* is split into kick and drift. *V* (*q*) is integrated with one integration point per element, both located at 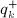. The kick is of infinitesimal duration.

Before proceeding with the treatment of constraints, the asynchronous procedure is expressed in terms of a sequence of kick and drift operators. Consider an explicit time element with discrete action *S*_*k*_ as illustrated in Figure 1. The resulting map is a function of the time step size *h* involving a simultaneous modification of momenta and coordinates. The time step is subdivided into an element of infinitesimal length *τ* and a second element of length *h* − *τ*. The first element contains the integration point of the potential energy and is called a kick, the second a drift. By introducing an additional support point *θ*_*k* + *τ*_, one is able to decouple the modifications of momenta and coordinates. The principle of stationarity leads to the kick operator


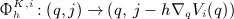
(4)

for the *i*-th potential and the drift operator


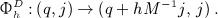
(5)

The latter solves the motion with assumed constant velocity.

Well-known time stepping schemes can be expressed using these notations. The velocity Verlet scheme is given by 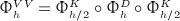. Its leapfrog representation becomes 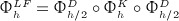. The symplectic Euler can be expressed as 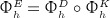.

**Figure 2:**
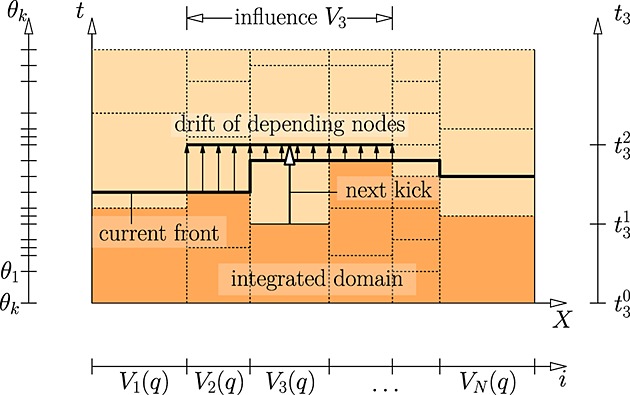
Illustration of a space–time front in one spatial dimension 30: The colored area is the space–time domain to be integrated (material space *x* × time *t*). The highlighted area is the domain that is already integrated. The deformed coordinates of all nodes are known at times *θ*^*A*^ defining the current front. It is generally not the boundary of the already integrated domain. This is because the most recently integrated space–time cells require the drift of all influencing nodes (which may be part of adjacent finite elements). Potential *V*_3_ is ‘kicked’ next at time 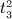 requiring the drift of surrounding nodes.

The asynchronous integrator described in Section 2 is a sequence of kick and drift operators. The subsequent sections are based on the following observations and assumptions:

- Every kick only modifies the momenta. The treatment of constraints should implement the same rule.
- A drift is applied node-wise. Only a few nodes must be drifted to apply a kick, see Figure 2. Application of a coordinate-dependent kick requires the determination of all nodes that affect the kick operator. Only these nodes must be drifted to the current kick time.
- The number of drifts per node is generally larger than the number of kicks during the total simulation. Compare, for example, the number of system times *θ*_*k*_ with the number of element times 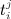, the latter being much smaller. Although the total number of kicks and system times *θ*_*k*_ are equal, each kick involves several nodal drifts. It is, therefore, recommended to formulate the drift as efficient as possible.
- Because of the existence of velocity dependent constitutive relations and velocity dependent constraint algorithms, one should use discrete velocities *v*_*k*_ instead of momenta *j*_*k*_ = *Mv*_*k*_.
- A priority queue decides which kick is applied at next. Each kick appears once in the queue that is kept sorted according to the next evaluation times. Because multiple kicks may have identical times, a secondary sort condition is required to make the ordering unique.
- Multiple types of kicks may exist. One type are kicks due to the strain energy as described in Section 2. Another type are external forces, for example, time-dependent loads that may be associated with individual time steps. Penalty forces are treated in the same way as restoring forces. Contact responses are individual kick operators in this article.

The resulting scheme is summarized in algorithm 1.


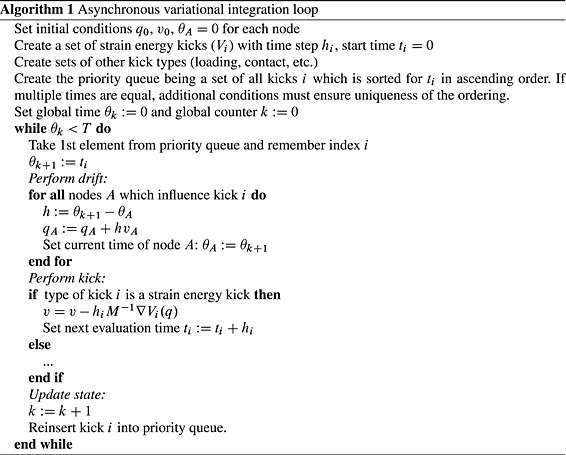


## 4. NODAL RESTRAINTS

### 4.1. Synchronous treatment of nonlinear constraints

Consider a mechanical system with coordinates *q* that are constrained by a set of smooth nonlinear holonomic equations summarized in the vector *g*,


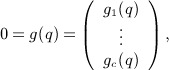
(6)

which Jacobian is of full rank, that is,


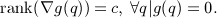
(7)

Because *g*(*q*) = 0 is satisfied at all times, this condition is equivalent to enforcing the hidden constraint


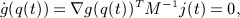
(8)

which is known as persistency condition. To ensure stability of a discrete algorithm, one must enforce both constraints at the same time. A symplectic-momentum scheme for the explicit velocity Verlet method is RATTLE 29, 31 given by


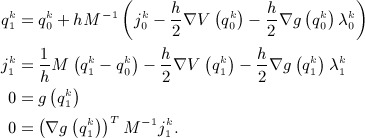
(9)

Given an initial condition (*q*_0_,*j*_0_) satisfying both sets of constraints, one iteratively computes the coordinates 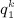 and multipliers 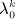 enforcing 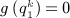. After that, 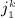 and multipliers 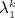 are computed from a linear system of equation enforcing 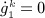.

The last operation is equivalent to a projection of the momenta 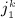 onto the constraint manifold with projection matrix 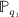



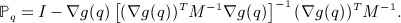
(10)

By using this notation, one can express RATTLE in terms of kick and drift operators, see Algorithm 2.


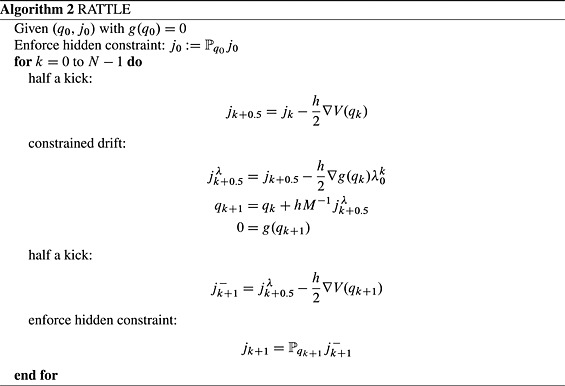


### 4.2. Asynchronous treatment of nodal restraints

Let the asynchronous discretization of nodal restraints be derived from RATTLE to ensure stability, symplecticity, and momentum preservation. A computational challenge of RATTLE in the asynchronous context is the constrained drift phase in Algorithm 2. For nonlinear constraints, the drift must be iteratively solved. If the constraints depend on multiple nodes, a coupling appears that may render the asynchronous procedure inefficient. It leads to a scheme wherein the drift of a single node requires the drift of all coupled nodes (and their dependent nodes in a recursive manner). The coupling of multiple nodes and the iterative nature are avoided by straitening the consideration to nodal restraint conditions.

Nodal restraints are linear constraint equations that depend only on the displacements of a single node *A*, that is,


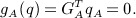
(11)

A finite element node may be subject to maximal three restraints. Restraints may be used to define simple boundary conditions, for example, sliding and rigid supports.

The objective is to find a strategy that eliminates the constraint *g*(*q*_*k* + 1_) and Lagrange multiplier 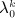 in the drift phase of algorithm 2. Observe that any constant motion satisfies a linear constraint equation if the initial coordinates and velocities are feasible. Hence, one must ensure that the velocities satisfy the persistency conditions at all times during the simulation. An efficient procedure is to apply any kick in such a way that the modification to the discrete momenta does not violate the hidden constraint. Then, the coordinate increments will satisfy the restraints after a drift phase as well. Each kick performs an additional projection to the momentum increment, that is,


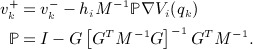
(12)

This is equivalent to a simultaneous execution of kick and projection in Algorithm 2. Assuming a diagonal mass matrix, the nodal mass can be canceled out of the fraction in the projection matrix. The presented approach, however, must ensure that the initial conditions satisfy the hidden constraints and that any other kick type (for example collisions) does not violate them.

When implementing the projection for nodal restraints, either the matrix [*G*^*T*^ *M*^−1^ *G*]^−1^ (together with *g*) or the projection matrix 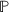 can be computed prior the simulation. The small size allows the storage of these matrices at the finite element nodes. For the first variant, one must save matrices of dimensions 0 × 0 to 3 × 3 (depending on the number of restraints); for the latter case the matrix 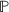 is 3 × 3 for all nodes.

## 5. ASYNCHRONOUS CONTACT SEARCH

### 5.1. The contact algorithm

During the simulation, the contact conditions must be satisfied at discrete points in time. For the solution algorithm one generally requires information on the activity of the constraints, the current residuum at the predictor configuration and eventually derivatives of the residuum. Given a predictor coordinate vector **x** one requires the following steps in a contact algorithm:

1. Global search: one has to identify the local element coordinates *ξ*^(*i*)^ on the contactor and target side for each given **x**. This is equivalent to the inverse mapping *φ*^−1^ with **x** = *φ*(**X**(*ξ*)). A brute force approach would compare all finite elements with **x** that unnecessarily increases numerical complexity. Instead, a global collision phase is carried out before the actual contact detection. In this phase, the set of possible collision candidates is reduced to a reasonably small number by means of very fast methods. These are potential contact pairs consisting of a contactor point and a target element/face being sufficiently close to the other.
2. Local search: the local search performs an accurate inside–outside test for the specified contact pair and finds the local coordinates *ξ*^(*i*)^ = *ξ*^(*i*)^(**x**).
3. Generation of constraint equations: for each positive detection, the discrete constraints are evaluated and temporarily stored (including discrete gradients, etc.).
4. Computation of response: given the active set of constraints, the response is computed.

Because the number of constraints may be large, it is advisable to integrate points 3 and 4 with the local detection phase: once a positive interpenetration of a node with some finite element is found, the response will be applied immediately before the next local intersection test takes place. This will reduce the number of temporary data objects.

### 5.2. Node-to-surface integration

For simplicity of notation, node-to-segment integration is used in this work. Therein, the numerical integration points on the contact boundary are coincident with the finite element nodes on the contactor's boundary. Then, the discrete closest point projection leads to the maps


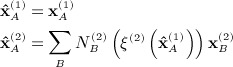
(13)

between points on the contactor boundary 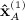 and on the target boundary 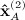 with finite element shape function *N*_*B*_ and local coordinate on the target segment *ξ*^(2)^. They are used to express the variations of the gap function and the glide path in the contact integral with respect to the virtual displacements *δ***u**. The contact constraints arising from impenetrability and friction are enforced at nodes *A*.

A general contact strategy is assumed, that is, no contact pairs are defined by the user a priori. Instead, all boundaries may be in contact with all bodies in the system. As a result, no assumption about the mortar side can be carried out to avoid overconstraints in a two-pass node-to-segment strategy. This would require a clear definition of contact pairs such that the algorithm can merge the integration points on the nonmortar side into the mortar integral. Overconstraint issues, however, do not exist in the asynchronous collision algorithm where the individual discrete contact constraints are applied sequentially and, thus, only a single constraint is considered in each step.

### 5.3. Global detection

Spatial partition schemes and hierarchical representations are often used to localize the regions where the actual collision appears or to delimit the domain where the exact collision test must be performed. Such representations approximate the topology of an object at different levels of detail. These include bounding volume hierarchies like sphere trees 32, 33, oriented bounding boxes-trees 34, axis-oriented bounding box (AABB) trees 35 and hierarchies of discrete orientation polytopes 36, as well as spatial partitioning such as octant trees 37, bucket trees 38, kd-trees 39, and position code algorithms 40–42, or totally different approaches such as spatial hashing 43 or image based methods 44.

**Figure 3:**
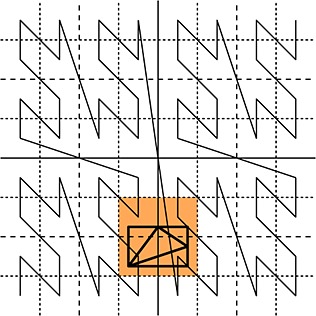
Contact detection by a position code by using a space-filling Lebesgue curve. Highlighted are the four cells to check.

Figure 3 illustrates a global contact detection by using an AABB as the bounding volume of a finite element (or finite element face) and utilizing a position code algorithm to subdivide the total space. The scheme subdivides the space into equisized cells that are numbered according to a space-filling curve.

Asynchronous collision detection is conceptually different from standard schemes:

- In synchronous time stepping schemes, the main question in collision detection is as follows: What are the finite element nodes that intersect with a given finite element or what are the nodes that are closest to a finite element face? In this case, the cells contain the appropriate finite element nodes. For a given target face, one determines all cells that are intersected by its bounding volume (AABB). For each cell, one determines the set of contained nodes that actually intersect with the AABB.
- In asynchronous schemes, the main question is: What are the AABBs that intersect with a given node?
  In this work, each cell stores the set of AABBs by whose it is intersected. Then, one computes the position code of a given node, finds the appropriate cell, and intersects the node with all AABBs of that cell. Compared with synchronous schemes, this implementation needs more memory for storing the AABBs at the cells (each AABB may be part of multiple cells). Furthermore, the position code update of moving AABBs is more complex than that of moving nodes.

### 5.4. Updating the spatial hierarchy during the asynchronous loop

The focus of asynchronous collision detection lies on updating the spatial data structures of global contact search with minimal effort. In synchronous schemes, the spatial hierarchy is updated for all nodes and elements at one time. In the asynchronous context, only a few nodes are affected by a single kick event or drift phase. In AVIs it is more crucial than in standard methods that the update of the data structures with respect to the affected nodes is a numerically cheap operation.

The priority queue is an ordered set of kick operators. Some of these kick operators are ‘contact elements’ each representing the collision of a single contactor node. Other kick operators represent the response because of the restoring forces of individual spatial integration points. The latter are associated with a set of affected finite elements. Spatial integration points of the strain energy may influence the degrees of freedom of a single finite element (if they are located in the element's interior) or of multiple finite elements (if they are located on the element's boundary, for example, in the nodes). Every finite element knows if it is subject to collision detection. In this case, it is associated with an AABB in the spatial data structure.

#### Strain energy kicks

Whenever a strain energy based kick appears, it applies a drift to all nodes that are influenced by the kick. It further checks if any associated finite element is subject to collision detection. In this case, all associated AABBs are updated with respect to the current coordinates. The coordinates of a single AABB are not necessarily synchronous. Assuming that the time step of strain energy kicks is generally very small, the spatial data structures can be considered being sufficiently accurate with respect to the target elements.

#### Collision kicks

Whenever a collision kick is taken from the priority queue, it first drifts the contactor node to the current time. The position code of the node is updated in the spatial hierarchy. Then one can perform a global collision detection for the associated node.

After finding the collision pair candidates in the global search phase, all involved nodes of a node-AABB-pair are drifted to the contactor node time. This will improve the accuracy of the local collision detection. Furthermore, it allows the accurate computation of the normal vector 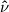, which is crucial for the collision integrator. In addition, the conservation of linear and angular momentum is only guaranteed, if the collision response is applied to the contactor point and the target element at the same system time and at the same spatial coordinate.

Subsequently, the local contact search is applied, that is, the local finite element coordinate *ξ*^(2)^ of the given node within the target element is obtained.

## 6. ASYNCHRONOUS COLLISION INTEGRATOR

### 6.1. Decomposition contact response

Consider a Hamiltonian system subject to a set of inequations collected in a constraint vector *g*, that is,


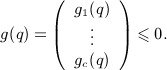
(14)

Inequalities require a nonsmooth setting. At any time, only a subset or no constraint may be active. As soon as a constraint *g*_*j*_ is activated, that is, 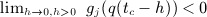 → *g*_*j*_(*q*(*t*_*c*_)) = 0, the trajectory of the generalized coordinates must be modified to stay feasible. The involved velocity changes generally are discontinuous (the trajectory is nonsmooth).

Parameterize the time with respect to a fictitious time *α*. Assume that the given set of inequations is active once during the considered time interval and that all constraints are active at the same time 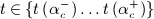. The action integral becomes


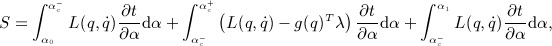
(15)

where the activation times *α*_*C*_ belong to the unknown variables. Variation yields


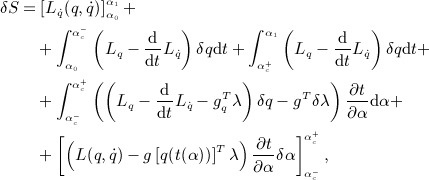
(16)

where the variation 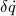 is transformed into *δq* by using integration by parts and where the variation of the (de)activation times *α*_*C*_ is determined from 
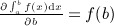
, 
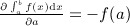
. The first term defines the initial conditions. The first two integrals yield the unconstrained equation of motion before and after the collision. The other terms lead to three equations that determine the collision time, the Lagrange multipliers, and the trajectory during the collision. For a detailed derivation see 24, 25.

**Figure 4:**
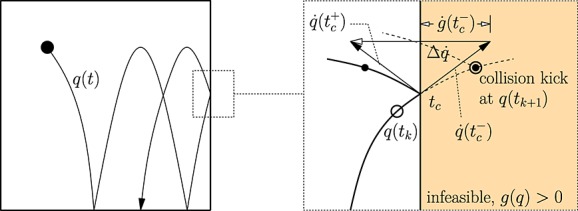
Collision of a particle under gravity. The trajectory *q*(*t*) is illustrated. The change of momentum at the boundary of the infeasible domain (or velocity, respectively) obviously is a nonsmooth process. The constraint is evaluated at the discrete coordinate *q*(*t*_*k* + 1_) because the *t*_*c*_ is generally unknown.

For further discussion a few simplifications are assumed: (1) inequalities are assumed to be active at discrete infinitesimal time steps, that is, 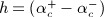 with 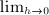; (2) each inequation *g*_*j*_ may become active at individual times *α*_*c*,*j*_ but assume that an active set *g*^*a*^ is established at a global activation time *α*_*C*_; these are all constraints 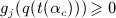. (3) Let it be sufficient to check the active sets at the end of each time step. The strategy is illustrated in Figure 4.

Algorithm 2 without the projection in its last step is applied to the infinitesimal time step at the collision. This yields an explicit collision integrator given by


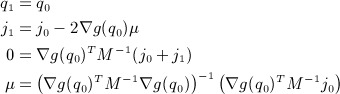
(17)

with Lagrange multiplier *μ*. It is equivalent to a projection by 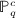 of the momentum ‘against’ the constraint manifold such that the constraint rate changes its sign, that is,


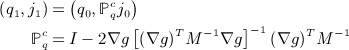
(18)

Due to the discrete nature of collision detection certain iterates may be infeasible. In such cases, the map would enforce unfeasibility, because it is not aware if a constraint is approaching from the feasible or infeasible domain. The constraint rate 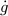 may be used to filter slightly violated and active constraints that will be feasible at the next time increment and that would be modified to stay infeasible by the collision integrator. Then the active set 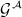 is


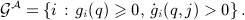
(19)

The collision integrator preserves the energy *E* (with ∇ *g* : = ∇ *g*(*q*_0_))


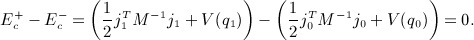
(20)

The approach is known as decomposition contact response (DCR) 25.

### 6.2. Normal contact

Given the local finite element coordinate *ξ*^(2)^ of contactor node *A* in the target element, one can assemble the discrete gap function gradient at node *A* through the variation


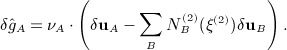
(21)

Therein, *ν*_*A*_ denotes the surface normal at the contactor node *A*, 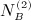 the finite element shape function of the target element, **u**_*A*_ the displacements of node *A*.

The collision response is obtained by DCR applied to a single active constraint. As in DCR, the collision time *t*_*c*_ of a single contact constraint is not resolved explicitly. But unlike in DCR, it is not necessarily the end of each time step uses for integrating the strain energy. Instead, the collision times may be chosen independently from the time step used in Equations (2) and (3). The selection of the time step between two collision detections is discussed in Section 7.

After finding a contact pair in the local contact detection, the gap rate is computed


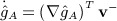
(22)

with discrete gap gradient 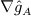 and discrete velocity vector **v**. The constraint is considered active if


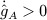
(23)

else the contact pair will be skipped.

Direct application of Equation (18) corresponds to the modification of velocities


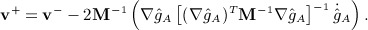
(24)

Assuming a diagonal mass matrix and nodal masses *m*_*A*_, this update can be computed efficiently. The gap gradient is sparse and, hence, only a few components of **v** are affected by the update. The asynchronous nature of the collision response leads to a sequential collision response involving simple equations with scalar Lagrange multipliers (17). This is contrary to synchronous algorithms where Equation (18) requires the solution of a system of linear equations because of coupling terms among individual contact constraints.

Application of a coefficient of restitution *κ* 25, 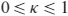, leads to


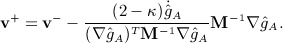
(25)

### 6.3. Normal contact with nodal restraints

If nodal restraints are defined, see Section 4, the computation of the contact response is not so easy because not only 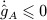 must be enforced. The contact projection must preserve the restraint conditions **G**^*T*^**u** = 0, Equation (11). An additional projection as in Equation (12) is not possible, because it does not obey the energy preservation properties of the contact response.

The system of Equations (17) is, therefore, extended by the restraints. Nevertheless, the problem can still be solved efficiently because at most three restraints can be defined per node and each subset of restraints influences only the displacements that belong to the same node. Furthermore, the equality constraints can be handled in the same manner as inequality constraints if the hidden restraint **G**^*T*^**v**^−^ = 0 is satisfied before the collision.

The Lagrange multiplier (17) becomes a vector consisting of the restraint multipliers *λ* and the contact multiplier *μ*


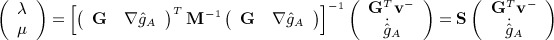
(26)

with decomposition of the inverse of matrix **S**


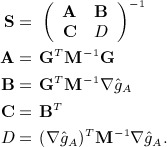
(27)

The matrix **S** always exists: the restraints are linearly independent by definition. Only the contact gradient may be linearly dependent on the restraints. This case is very unlikely. It can be checked easily and may only appear in erroneous generated meshes.

The update of the velocities is then computed from


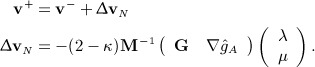
(28)

To solve the system of equations one has to find a simple expression for


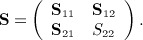
(29)

**S** is given by


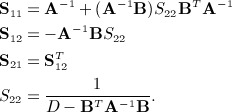
(30)

By using these definitions and assuming that the restraint rates are kept zero during the simulation, the Lagrange multipliers simplify to


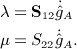
(31)

When implementing the computation of the multipliers, one has to provide fast mappings between node indices, indices of global degrees of freedom and the local DOF indices at nodes. The mass is diagonal and constant for all DOFs of the same node. The blocks of matrix **A**^−1^ (without mass, that is, (**G**^*T*^**G**)^−1^) and the blocks of matrix **G** are stored at each node. During each collision response, one first computes the vector **B** and the scalar *D*. Then, the vector


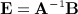
(32)

is computed and temporarily stored. The scalar *S*_22_ is computed by


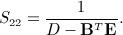
(33)

For the multipliers, one obtains


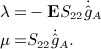
(34)

The product (32) can be obtained efficiently by using the block structure of matrix **A**


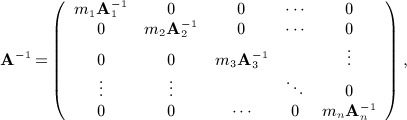
(35)

wherein 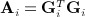 are precomputed constant symmetric, positive definite matrices of maximum dimension 3 × 3 per node *i* and *m*_*i*_ denotes the respective nodal mass. For the computation of **E**, one does not need all nodes, because the sparse vector **B** only contains elements that belong to the nodes that are affected by the considered collision. For the construction of **B** and **E**, it is helpful to determine an ordered set of the involved nodes. Then one iterates through the set of involved nodes and adds three components (three DOFs per node) to **B** and **E**. In fact, it is not necessary to generate **B** at all. The contributions of each node can be added to **E** and 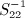 during the loop through the involved nodes set.

### 6.4. Coulomb friction

The collision response requires the computation of the normal velocity increment Δ**v**_*N*_, Equation (28). The tangential velocity increment is computed from the decomposition


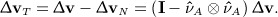
(36)

Hence, no assumptions on the smoothness of the contact surface are required for the computation of the tangential basis. In a predictor step, the yield surface of the friction law is ignored and the relative velocity along the normal and tangential direction is enforced being zero, that is,


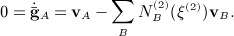
(37)

These are three additional constraints that enforce a zero relative velocity of both contacting material points, one constraint along the direction of each Cartesian axis. The gradient matrix 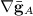 is assembled from the variation


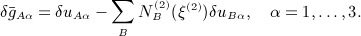
(38)

The computation of the corresponding velocity increment 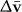 is similar to the normal response Δ**v**_*N*_, but this time there are three instead of a single contact constraint. In the presence of nodal restraints, the solution involves the following steps:


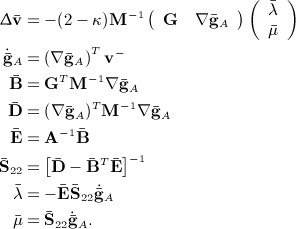
(39)

A predictor of the tangential velocity increment can be obtained by


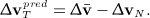
(40)

The friction law is applied to the tangential velocity by checking the Coulomb yield surface for each predicted nodal tangential traction. The nodal contact tractions are the momentum changes


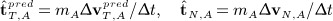
(41)

with node index *A* and nodal mass *m*_*A*_. Obviously, the nodal mass and the time step length can be eliminated from the Coulomb yield condition and the velocity increments can be used directly. The tangential velocity change for node *A* is then


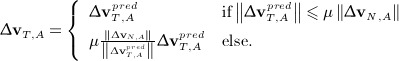
(42)

The post collision velocity is obtained from


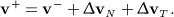
(43)

## 7. TIME STEP SELECTION

### 7.1. Sequential synchronous collisions

The asynchronous collision response can be used to improve explicit synchronous contact algorithms. One reason for the inefficiency of those algorithms is the factorization of the matrix 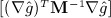 in Equation (18) when multiple contacts are active. Although the matrix itself is very sparse, its inverse may be dense. Furthermore, its dimension is the number of nodes being in contact. The computation of the projection is, therefore, computationally expensive. The contact gradients 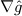 change with time and, therefore, the factorization must be repeated at each time step. Furthermore, the matrix may be singular if spatial discretizations equivalent to the two-pass node-to-segment integration are used.

The sequential procedure, on the other hand, may be less accurate in regions with complex geometries. Using a smaller time step may, however, improve the accuracy. A sequential response is equivalent to the collision procedure presented in 24 if the actual collision times *α*_*C*_ are not accurately determined and are set to the discrete times *θ*_*k*_.

A synchronous sequential procedure may be more efficient than a completely asynchronous collision response. This is because the data structures used in global collision detection must be updated only at synchronous times. The number of these times is much smaller than in the asynchronous setting, but then the complete structure is affected instead of a small spatial region.

### 7.2. Asynchronous constant time steps

Herein, the time step of the collision kick of a node *A* is chosen to be the minimal critical time step of the adjacent finite elements.

### 7.3. Asynchronous adaptive time step selection

Asynchronous integration targets at problems with spatially varying mesh densities. Then there may exist regions on the contact boundaries with very fine meshing and other domains with very coarse meshing. In such cases, the time step of a synchronous collision response is tied to the smallest mesh size on the boundary. A time step too large may invoke interpenetrations that are not detected. A situation can be improved by assigning smaller time steps to surface patches of smaller size. Furthermore, the time step sizes can be arbitrarily chosen. There exists no critical time step to the collision response, whereas the only restriction is given by the accuracy of the contact detection.

Define a representative quantity for the ‘length’ *l*_*A*_ of a finite element node *A*, for example,


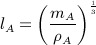
(44)

with nodal mass *m*_*A*_ and nodal mass density *ρ*_*A*_. Then every single collision response may be associated to an individual kick event *i* with kick time 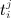 that is kept in the priority queue of the asynchronous integrator. After each response, the next kick time is computed


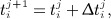
(45)

and the kick is reinserted into the priority queue. The time step is


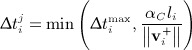
(46)

such that the time step length depends on the size of the node and the current absolute nodal velocity.

Ideally, a quantity for the relative velocity to close contact candidates should be chosen, but this information is not available in most cases. By using a two-pass node-to-segment strategy, however, a conservative estimation can be obtained: if two nodes with distance Δ*x* approach each other with velocities *v*_*A*_ and *v*_*B*_, then the collision time will be at least 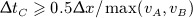. It is sufficient, when the faster node handles the collision detection. In a two-pass strategy, where both nodes are associated to collision detection times, it is irrelevant which node has the faster velocity.

When choosing the constant *α*_*C*_, one must ensure that the next collision detection must take place before the considered node may penetrate the target too deep (or before the target penetrates the contactor too deep when taking the characteristic length of the contactor side).

## 8. NUMERICAL EXAMPLES

### 8.1. Two elastic bars

**Figure 5:**
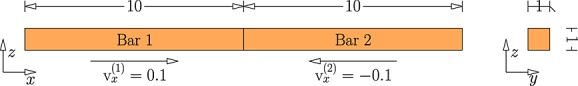
Impact of two bars.

This example is excerpted from 25, 45. A longitudinal impact of two elastic bars is considered, see Figure 5. The geometry of each bar is given by *L* = 10, *H* = *B* = 1. A linear elastic material (with small strains) is chosen with Young's modulus *E* = 1, Poisson's ratio *ν* = 0, and mass density *ρ* = 1. The impact is full elastic. Before the collision, the velocities are *v*^(1)^ = − *v*^(2)^ = 0.1. The bar tips should remain in contact for *t* < 20.

The asynchronous integrator is used with a time step ratio *β* = 0.5 related to the critical time step. The step size parameter for the collision kicks is given by *α*_*C*_ = 0.75 with maximum collision step time being the average critical time step. As a reference solution serves velocity Verlet where DCR is applied to the midpoint of each time step (this will improve the accuracy to the original formulation being applied between two time steps). The time series history variables are determined for both at certain points at time. The total simulation time is *T* = 50; the number of save intervals is 5000. When measuring the required CPU time, the evaluation of history variables is turned off, because it may affect the performance.

For both integrators, the same contact detection algorithms are applied. For velocity Verlet, the faster sequential response, see Section 7, is used for a fair comparison. A general contact methodology is applied, that means all bounding faces are subject the collision detection. Although the normal vectors at the corners and edges may not be accurate, the direction of the response is computed nearly accurately: the motion of the two bars is parallel to the longitudinal axis.

The mesh of the first bar consists of 5*n* × *n* × *n* eight-noded brick elements. The mesh size parameter *n* controls the number of elements per side. The element sizes along the *y* and *z* direction are uniform. Along the *x* axis, the position of nodes is chosen to be


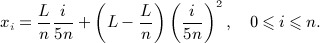
(47)

**Figure 6:**
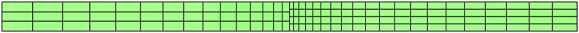
Impact of two bars: mesh for *n* = 3.

That means, the smallest elements are located at the contact interface. Along the *x* axis, the element size grows linearly. To enforce a nonconforming mapping at the contact interface, the right bar is discretized by 5(*n* + 1) × (*n* + 1) × (*n* + 1) elements with equivalent node positions, see Figure 6.

**Figure 7:**
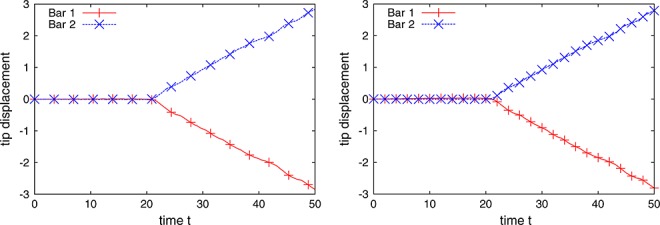
Impact of two bars: tip displacements over time. Left: Verlet. Right: asynchronous variational integrators. *n* = 4.

**Figure 8:**
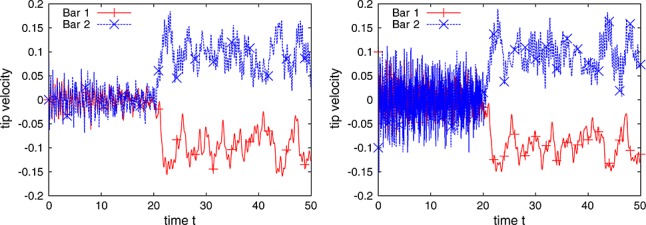
Impact of two bars: tip velocities over time. Left: Verlet. Right: asynchronous variational integrators. *n* = 4

The tip displacements for mesh size parameter *n* = 4 are illustrated in Figure 7. The used time steps are *h* = 0.0174 for Verlet and for AVI *h*_min_ = 0.0129, *h*_max_ = 0.0485, *h*_average_ = 0.0264. The tip velocities are presented in Figure 8. To get single quantities for the tip nodes, the values of all nodes located at the tip are averaged. The displacements are in good agreement.

There exist spurious oscillations in the velocities during the persistent impact phase. Such were also reported by others applying explicit collisions 25. They result from the explicit representation of the discrete potential action *V*_*d*_. The oscillations can be reduced by decreasing the time step length. The spurious oscillations are greater if the collisions are applied asynchronously.

The reason for the greater oscillations lies in the asynchronicity of the time stepping scheme. The time steps between two contact kicks and the time steps between two restoring force kicks are not directly related. Furthermore, the used time step between collisions is larger than the one used for the strain energy. The asynchronicity has the interesting effect that a spatial point under consideration may have a discrete velocity that changes rapidly, but these changes are only visible on a micro time scale, for example, between two close system times *θ*_*k*_ and *θ*_*k* + 1_ as in Figure 2. The difference between *θ*_*k*_ and *θ*_*k* + 1_ is mostly significantly smaller than the actual time step between two collisions *h*_*c*_. The velocity vector shown in Figure 8 is computed from the discrete momenta by *v*_*k*_ = *M*^−1^*j*_*k*_. Hence they can be interpreted as – losely spoken – purely ‘numerical’ quantities required to enforce continuity of the piecewise linear trajectory of *q*(*t*), see 30. A ‘better’ approximation of the tip velocity would be to take an average increment of the displacements over the interval length of a contact time step, that is, *v*_*k*_ = (*q*(*θ*_*k*_ + *h*_*c*_) − *q*(*θ*_*k*_)) / *h*_*c*_, which reduces the oscillations significantly.

**Figure 9:**
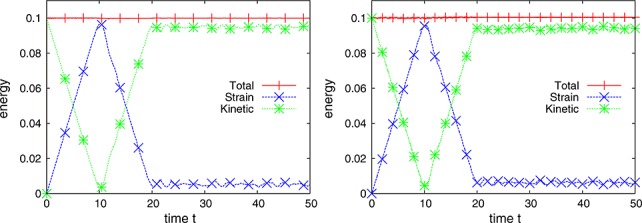
Impact of two bars: energy balance over time. Left: Verlet. Right: asynchronous variational integrators. *n* = 4.

The energy balance is presented in Figure 9. Both algorithms nearly preserve the total energy. There is a very small energy drift in the asynchronous integrator, however, that seems to be subject to the asynchronicity of the strain energy evaluations.

**Figure 10:**
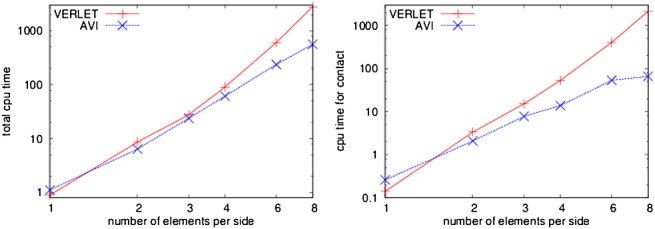
Impact of two bars: CPU time for different mesh sizes. Left: total CPU time. Right: CPU time due to contact algorithm.

The CPU times are presented in Figure 10. It illustrates the total CPU time and the time that was spent by the contact algorithm. The latter is measured as the difference of the total CPU times of two simulations, one with and one without contact. Obviously, the numerical cost grows significantly slower in the asynchronous case.

### 8.2. Elastic block sliding on rigid obstacle

This example serves to test Coulomb friction and nodal restraints. A block with dimensions 1 × 1 × 1 is sliding on a rigid plane subject to gravitation. The block is discretized by 3 × 3 × 3 eight-noded brick elements. The obstacle is discretized by 5 × 5 × 1 elements and has the dimension 5 × 2 × 0.2. All nodes of the basement are fixed through nodal restraint conditions. A St. Venant material is used (linear elastic with finite strains) with Young's modulus *E* = 100, Poisson's ratio *ν* = 0.2, and mass density *ρ* = 1. The body is subject to a constant vertical body force *F* = 1. The initial horizontal velocity is *v*_*x*_ = 1. The Coulomb parameter of friction is *μ* = 0.5. The total simulation time is *T* = 3. The time step ratio related to the critical time step is *β* = 0.5. The step size parameter for the collision kicks is given by *α*_*C*_ = 0.5 with maximum collision step time being the average time step.

**Figure 11:**
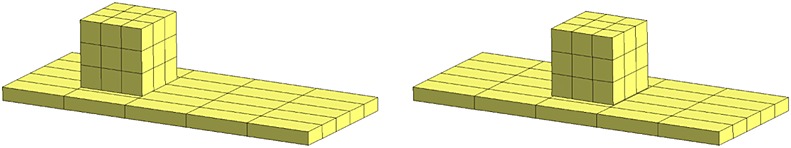
Sliding block: start and end geometry.

**Figure 12:**
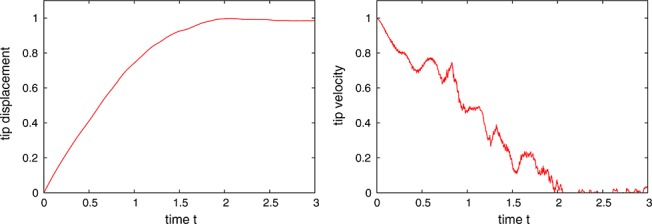
Sliding block: displacement and velocities over time. Left: displacement. Right: velocity.

**Figure 13:**
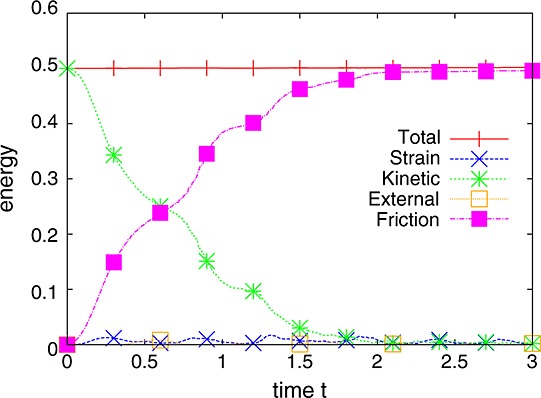
Sliding block: energy balance over time.

Figure 11 illustrates the geometry at the beginning and at the end of the simulation. Figure 12 presents the horizontal displacements and velocities at the block's bottom. A single value of the displacements is obtained by averaging the nodal values at the bottom surface. The results are in good agreement with the analytical solution of a rigid block: the displacements describe a parabola with end displacement *u*_*x*_ = 1, whereas the velocity decreases linearly until time *t* = 2. Figure 13 presents the energy balance. The energy dissipated by the friction grows until almost no energy is left in the system. The total energy is nearly preserved by the algorithm.

### 8.3. Elastic block rolling on rigid obstacle

**Figure 14:**
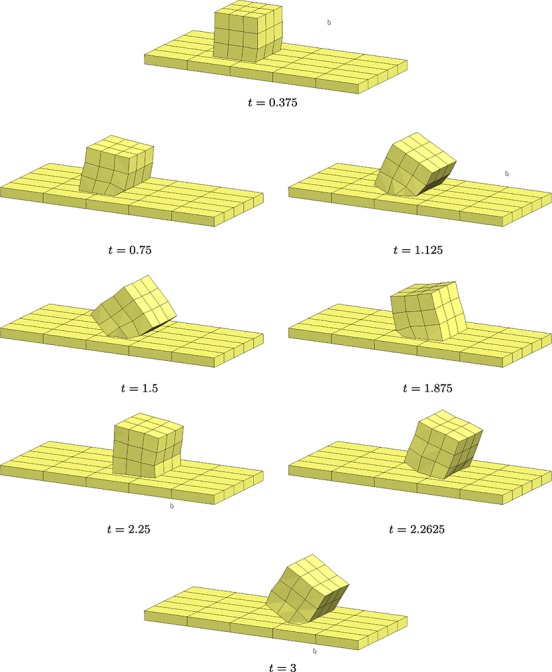
Soft sliding block. Geometry at different times.

**Figure 15:**
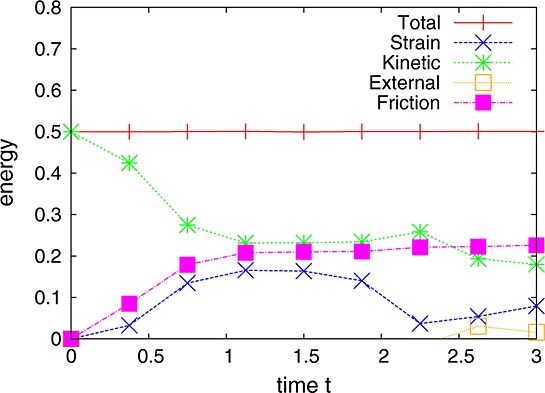
Soft sliding block: energy balance over time.

The material parameters of the last example are changed to *E* = 10 and *ν* = 0.2. Because of the reduced stiffness, the block starts to roll on the interface. Some configurations are presented in Figure 14. The energy balance is shown in Figure 15.

### 8.4. Block assembly

**Figure 16:**
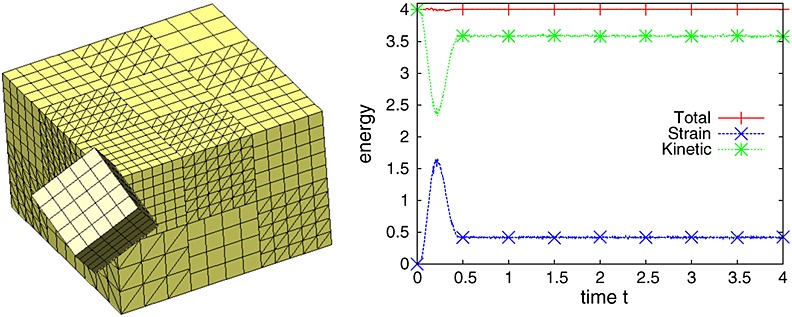
Block assembly: geometry and energy over time.

This example illustrates the asynchronous collision procedure applied to a rather complex problem. An assembly of 18 cubes is hit by another moving cube. The geometry of the initial frame is presented in Figure 16. All cubes are of dimension 1 × 1 × 1. The hitting cube is rotated around its center by the axis 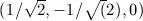 and the angle *π* / 4. Its center is defined by ( − 0.5, − 0.5,2), whereby the origin (0,0,0) is defined in one of the bottom corners of the block assembly. Every cube is discretized by either first-order tetrahedra or first-order hexahedra on a regular grid, each cube with different element sizes. Stabilized nodal integration is used to integrate the strain energy. A St. Venant material is used (linear elastic with finite strains) with Young's modulus *E* = 1000, Poisson's ratio *ν* = 0.3, and mass density *ρ* = 1. The initial velocity of the hitting cube is 2 in *x* and *y* direction.

**Figure 17:**
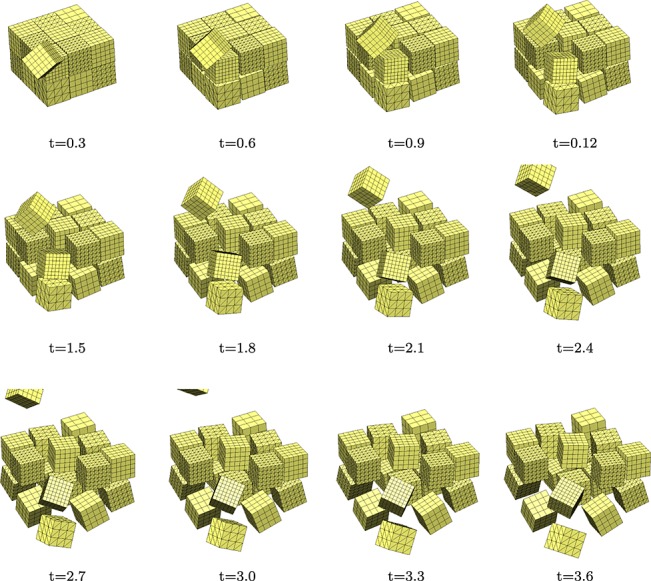
Block assembly: geometry at discrete times.

The total simulation time is *T* = 4. The time step ratio related to the critical time step is *β* = 0.5. The step size parameter for the collision kicks is given by *α*_*C*_ = 0.25 with the maximum collision step time being twice the average time step. The minimal critical time step in the system was identified as 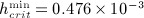, the maximum critical time step as 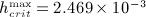, and the average being 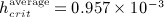. Figure 16 illustrates the energy over time. The total energy error does not exceed 1*%*. Figure 17 presents the geometry at various times during the simulation.

When analyzing the geometries at discrete times, small interpenetrations can be observed. These have three reasons:

- The *spatial density* of the integration points in the contact search is too small. During the first impact between the hitting cube and the block assembly, one cube is strongly deformed at its corner, the other in the center of its face. Some interpenetration can not be detected, because subsequent collisions between contactor edges and target elements are not found by the contact search. To improve the accuracy, one needs to add more integration points to the contactor surface or apply additional search strategies for edges and faces, see for example 25. The search strategy presented therein can be easily incorporated by adding additional collision kicks by using the alternative contact detection.
- *Cumulative effects*. The algorithm tries to prevent collisions by changing the velocity. The momentum change is applied at times, where already a collision was detected. Even if a correct velocity change was computed, subsequent events may increase the interpenetration until the next collision response takes place. A predictor–corrector algorithm could improve this, but is not efficient in explicit analysis, in particular in asynchronous simulation. In 25 the velocity change was combined with a non-symplectic coordinate change that tries to eliminate existing penetrations. Such a strategy, however, greatly increases the algorithmic complexity in asynchronous integration.
- *Too large collision time steps*. One reason for the superiority of asynchronous collisions with respect to CPU time is that less contact detections take place. In this example, 24884 spatial integration points for the strain energy and 3752 spatial integration points for the contact integral are used. During the simulation, 238,944,839 strain energy kicks and 7,837,942 collision kicks were performed. This is equivalent to average time steps of 0.416 × 10^−3^ for the strain energy and 1.915 × 10^−3^ for the collision detection.

## 9. CONCLUSIONS

Asynchronous integration is designed for finite element meshes with varying mesh densities. Explicit AVI performs a sequence of drifts with constant velocities. The velocities of individual spatial points are modified as asynchronous discrete times by kick operators. The treatment of nonlinear constraints must be expressed through a discontinuous modification of the velocities.

Nodal restraint conditions are expressed through a projection of the velocities onto the constraint manifold. It can be performed efficiently and solves linear restraints accurately. The solution of momenta and coordinates is identical to the momentum-symplectic RATTLE scheme.

In synchronous contact detection, the smallest surface patch decides on the time step size between two collision detections. In the presence of small and large finite elements in the same mesh, the frequency at which each element is tested on collision can be adopted to the element size. This approach assumes that the collision response does not affect the critical time step, which is true for DCR. Furthermore, DCR only changes the momentum and, thus, can be interpreted as a kick event in the asynchronous procedure. An additional correction of the displacements that directly eliminates spurious interpenetrations seems to be very difficult in AVI. A large coefficient of restitution may, therefore, lead to rather large penetrations, because small constraint violations are accumulated.

Decomposition contact response was applied to AVI termed asynchronous collision response. The treatment of collisions includes inelastic impacts, Coulomb friction, and the presence of nodal restraints. All of them can be solved by very small systems of equations because only one constraint is treated at one time. Hence, the algebraic solution is very fast compared with synchronous contact where the coupling of degrees of freedom leads to large matrices to be factorized. The asynchronous treatment further eliminates the appearance of over-constrained configurations where the two-pass node-to-element integration generates two equivalent constraints. The asynchronous collision response provides three interpretations in the implementation: (1) an adaptation of individual time steps for each boundary node with respect to accuracy (velocity and element sizes); (2) a fixed step size strategy where the individual step sizes are adjusted to the critical time step of adjacent finite elements; and (3) synchronous collisions where each contact constraint is processed sequentially. Numerical examples verified the accuracy and efficiency of the asynchronous collision response method. Normal contact and frictional contact were tested. Energy and momentum preservation was excellent. Furthermore, the potential to save computing time turned out to be even greater than in asynchronous integration of the strain energy. This was because the asynchronous procedure allows the contact detection being less frequent than the evaluation of the strain energy.

Possible research directions are to study the convergence, to use higher-order mortar methods that improve the spatial accuracy, and to invent global collision detection algorithms that are better suited for the asynchronous methodology.
